# Secretome Identifies Tenascin-X as a Potent Marker of Ovarian Cancer

**DOI:** 10.1155/2015/208017

**Published:** 2015-05-18

**Authors:** Marianne Kramer, Sandra Pierredon, Pascale Ribaux, Jean-Christophe Tille, Patrick Petignat, Marie Cohen

**Affiliations:** ^1^Department of Gynaecology Obstetrics, Faculty of Medicine, 1211 Geneva 14, Switzerland; ^2^Division of Clinical Pathology, HUG, 1211 Geneva 14, Switzerland

## Abstract

CA-125 has been a valuable marker for the follow-up of ovarian cancer patients but it is not sensitive enough to be used as diagnostic marker. We had already used secretomic methods to identify proteins differentially secreted by serous ovarian cancer cells compared to healthy ovarian cells. Here, we evaluated the secretion of these proteins by ovarian cancer cells during the follow-up of one patient. Proteins that correlated with CA-125 levels were screened using serum samples from ovarian cancer patients as well as benign and healthy controls. Tenascin-X secretion was shown to correlate with CA-125 value in the initial case study. The immunohistochemical detection of increased amount of tenascin-X in ovarian cancer tissues compared to healthy tissues confirms the potent interest in tenascin-X as marker. We then quantified the tenascin-X level in serum of patients and identified tenascin-X as potent marker for ovarian cancer, showing that secretomic analysis is suitable for the identification of protein biomarkers when combined with protein immunoassay. Using this method, we determined tenascin-X as a new potent marker for serous ovarian cancer.

## 1. Introduction

Ovarian cancer is the ninth most common cancer among women and is responsible for more fatalities than any other disease of the reproductive system. In 2013, it is predicted that 22,240 women will get diagnosed with ovarian cancer and 14,230 of these women will succumb to the disease in the United States, making a mortality rate of 63.9% [1]. Ovarian cancer is classified in four stages, with stage I being contained in the ovary and stage IV metastasizing into the peritoneal cavity. Each increasing stage is associated with a poorer prognosis and a decreased 5-year survival rate, with only 18% of patients diagnosed at stage four surviving five or more years [1]. If ovarian cancer is detected before it metastasizes outside of the ovary, there is a 92% 5-year survival rate; however, only 15% of women are diagnosed before metastasis [1]. Although the exact cause and cell of origin of ovarian cancer are dependent on the type of ovarian cancer, it is believed that serous ovarian cancer, the cell of origin of serous ovarian cancer may involve cells from the fallopian tube (for review see [2]).

Currently, the most common imaging techniques used to diagnose ovarian cancer are PET, MRI, and CT scans as well as transvaginal ultrasounds, but these procedures are unable to distinguish between benign and malignant ovarian diseases when confined to the ovary at early stages. Diagnoses are generally made by a transvaginal ultrasound accompanied by CA-125 serum testing, which is currently the only FDA approved molecule for monitoring recurrence [3]. There is an increasing need for protein markers to detect early stages of ovarian cancer before metastasis. Recently, proteomic techniques have been shown to be effective in identifying disease biomarkers [4]. However, it is still to be determined whether these molecular screening methods will lead to an overall decrease in mortality; there is some occurrence of surgical complications due on false positives because of the low specificity of markers [5]. It is worthwhile to mention that a high sensitivity of 99% would still require 25 abdominal surgeries to uncover 1 case of cancer [6]. CA-125 is an antigen found on the surface of ovarian epithelial cells and is absent in normal adult ovaries. This marker is seen in 50% of stage I ovarian cancer patients and more than 90% of patients with advanced stages [7]. It is currently the most common serum marker used for ovarian cancer diagnosis with a positive predictive value of less than 10% [8]. Wide arrays of techniques are available for the identification of proteins in serum, making proteomic analysis of diseases more widely available. Based on promising proteomic results, it is possible that high-throughput proteomic profiling will play an important role in the early detection of ovarian cancer [9].

We recently found more than 60 proteins that were differentially secreted compared to control cells thorough proteomic analysis of a patient with high-grade serous adenocarcinoma (submitted for publication). The goal of this study was to identify novel ovarian cancer serum markers by examining correlations between these previously identified proteins and CA-125 in a single patient study with FIGO stage IIIC serous adenocarcinoma.

## 2. Materials and Methods

### 2.1. Ethics Statement

The departmental ethics committee of maternity and pediatrics, University Hospital of Geneva, has approved this research. Informed written consent was obtained from all patients before their inclusion in the study.

### 2.2. Purification of Cancer Cells

Ascites were centrifuged at 600 g for 8 minutes. The cell pellet was resuspended in Hanks Balanced Salt Solution (HBSS, Gibco, Invitrogen, Basel, Switzerland) containing 25 mM HEPES (Gibco, Invitrogen, Basel, Switzerland) and 0.05 mg/mL gentamicin (Invitrogen, Basel, Switzerland) and centrifuged at 600 g for 8 minutes. The resulting pellet was resuspended in HBSS-HEPES-0.05 mg/mL and filtered through a 100 *μ*m mesh (BD Biosciences, San Jose, USA). The filtrate was resuspended in Dulbecco's Modified Eagle Medium (DMEM, Sigma Aldrich, St. Louis, MO, USA) containing 10% fetal bovine serum (FBS, Biochrom AG, Oxoid AG, Basel, Switzerland) and 25 *μ*g/mL plasmocin (InvivoGen, San Diego, CA, USA). This cell suspension is loaded onto a Percoll (GE Healthcare, Zurich, Switzerland) gradient, consisting of 4 layers of Percoll diluted in HBSS at 10%, 30%, 40%, and 70%, and centrifuged at 1200 g for 20 minutes. To eliminate blood cells, a Percoll gradient is performed containing 4 layers at different Percoll concentrations diluted in HBSS (3 mL of 10%, 3 mL of 30%, 2.5 mL of 40%, and 7.5 mL of 70%). Then, the cellular ring between layers 40% and 20% of Percoll was collected, diluted in DMEM, and centrifuged at 600 g for 8 minutes. The pellet was resuspended and 5.0 × 10^5^ cells were counted and seeded in a 3 cm dish. Cells were then characterized by PCR (cytokeratins 8, 18, and 19, HE4, and Pax8) and by immunocytochemistry (cytokeratins 7, 18, and 19, vimentin, and p53).

### 2.3. Purification of Benign and Control Cells

Ovarian tissue was digested with 4 mg/mL dispase (Gibco, Invitrogen, Basel, Switzerland) in HBSS-HEPES (filtered on 22 *μ*m) containing 1 *μ*g/mL DNase (Roche, Diagnostics GmbH, USA) for 30 minutes at 37°C. Ovarian tissue and supernatant were put in 10 cm dish and tissue was scrubbed with a scalpel. The supernatant was then collected, neutralized with 5% FBS, filtered through a 100 *μ*m mesh (BD Biosciences, San Jose, USA), and centrifuged at 2200 rpm for 8 minutes. The resulting pellet was resuspended in DMEM 10%, FBS 0.05 mg/mL, gentamicin 25 *μ*g/mL plasmocin, and 5.0 × 10^5^ cells were counted and seeded in a 3 cm dish.

### 2.4. Cell Culture

Ovarian cancer (from ascites), benign, and control cells were cultured in DMEM medium containing 10% FBS, 0.05 mg/mL gentamicin, and 25 *μ*g/mL plasmocin for 72 h for RNA extraction. For study of secreted proteins, cells isolated from the same patient, at four different times, were incubated in complete medium for 24 h, followed by 48 h in culture medium without FBS. Then, supernatants were collected and kept at −20°C until preparation for analysis.

### 2.5. Proteomic Analysis

Proteomic analyses were performed as previously described [10].

#### 2.5.1. Supernatant Concentration

Supernatants were concentrated on Vivaspin 500 3 kDa (GE Healthcare, Zurich, Switzerland) and protein concentrations were determined by Bio-Rad assay.

#### 2.5.2. Liquid Digestion of Proteins

Ten micrograms of proteins from each sample was dissolved in 100 *μ*L of 6 M urea, 50 mM Ammonium Bicarbonate (BA) solution and incubated at 37°C for 30 min. Then, 2 *μ*L of 50 mM was added and the mixture was incubated at 37°C for 1 h.

Addition of 2 *μ*L of 400 mM iodoacetamide to the proteins mixture and incubation for 1 h at room temperature in the dark with shaking allow alkylation reaction.

The samples were diluted 3X in 50 mM BA before addition of 5 *μ*L of a 200 ng/*μ*L solution of trypsin porcine (sequence grade modified, Promega) in 50 mM BA. The mixture was incubated overnight at 37°C. Finally samples were desalted with a C18 microspin column (Harvard apparatus, Holliston, MA, USA), dried in a SpeedVac, and redissolved in 5% CH3CN/0.1% FA before LC-ESI-MS/MS analysis.

#### 2.5.3. Peptide Fragmentation Sequencing

LC-ESI-MS/MS was performed on a linear trap quadrupole (LTQ) Orbitrap Velos (Thermo Electron, San Jose, CA, USA) equipped with a NanoAcquity system (Waters). Peptides were trapped on a homemade 5 *μ*m 200 Å Magic C18 AQ (Michrom) 0.1 × 20 mm precolumn and separated on a commercial 0.075 × 150 mm Nikkyo (Nikkyo Technology) analytical nanocolumn (C18, 5 *μ*m, 100 Å). The analytical separation was run for 65 min using a gradient of H2O/FA 99.9%/0.1% (solvent A) and CH3CN/FA 99.9%/0.1% (solvent B). The gradient was initially per 0-1 min 95% A and 5% B and then to 65% A and 35% B for 55 min and 20% A and 80% B for 65 min at a flow rate of 220 nL/min. For MS survey scans, the orbitrap (OT) resolution was set to 60000 and the ion population was set to 5.0 × 10^5^ with an *m*/*z* window from 400 to 2000. For protein identification, up to eight precursor ions were selected for collision-induced dissociation (CID) in the LTQ. The ion population was set to 1.0 × 10^4^ (isolation width of 2 *m*/*z*) while, for MS/MS detection in the OT, it was set to 1.0 × 10^5^ with an isolation width of 2 *m*/*z* units. The normalized collision energies were set to 35% for CID.

#### 2.5.4. Protein Identification

Peak lists were obtained from raw orbitrap data using the EasyProtConv conversion tool from the EasyProt software platform [11]. The peaklist files were searched compared to the SwissProt database (release 15.10 of September 21, 2011) using Mascot (Matrix Sciences, London, UK). Human taxonomy (20323 sequences) was specified for database searching. The parent ion tolerance was set to 10 ppm. Variable amino acid modifications were oxidized methionine and carbamidomethyl cysteine. Trypsin was selected as the enzyme, with one potential missed cleavage, and the normal cleavage mode was used. The mascot search was validated using Scaffold 3.6.5 (Proteome Software, Portland, OR). Only proteins matching with two different peptides with a minimum probability score of 95% were considered as correctly identified.

### 2.6. Immunohistochemistry

Ovarian healthy (*n* = 12) and cancer (*n* = 8) tissues were rapidly washed with 0.1 M phosphate buffered saline (PBS) at pH 7.4 and fixed for 4–12 hours in 4% buffered formalin at 4°C. The specimens were then dehydrated in ethanol and embedded in paraffin wax. Serial sections of tissue were deparaffinized and rehydrated through graded ethanol. Antigen retrieval was performed by microwave pretreatment in 10 mmol/L citrate buffer (pH 6.0) for 5 minutes four times, followed by cooling in a cold water bath. Nonspecific binding was blocked with 3% (v/v) bovine serum albumin (BSA) in PBS for 30 minutes at room temperature. The sections were incubated with anti-human tenascin-X (diluted in 3% BSA-PBS, H-90, Santa Cruz Biotechnology, Labforce, Nunningen, Switzerland, or AF6999 from R&D) or with control IgG (sc-2027, Santa Cruz Biotechnology, Labforce, Nunningen, Switzerland) overnight at 4°C. Sections were then washed with PBS and incubated with goat anti-rabbit or rabbit anti-sheep IgG-HRP (dilution 1/500) for 1 hour. After washing, sections were stained with diaminobenzidine (DAB) chromogen system (Dako, Baar, Switzerland). The stained tissue was scored independently by 2 experts. The intensity of staining was scored as absent (0), weak (1), moderate (2), and intense (3).

### 2.7. ELISA Assay

The level of tenascin-X in serum was measured in healthy patients (*n* = 7), patients with benign disease of ovary (*n* = 8), and high-grade serous ovarian patients (*n* = 13) by ELISA assay (Cusabio, Wuhan Huamei Biotech, China) following manufacturer's protocol.

### 2.8. Western Blot Analysis

Most abundant proteins from serum were depleted using Top2 depletion kit (Pierce, Life Technologies, Zug, Switzerland). Circulating proteins were then fractionated by 10% SDS-PAGE and transferred to nitrocellulose membrane for immunoblot analysis using mouse monoclonal anti-tenascin-X antibodies. Secondary antibodies were anti-rabbit-HRP. All antibodies were diluted in 5% PBS-milk and incubated overnight at 4°C for primary antibodies and 1 hour at room temperature for secondary antibodies. Specific signal was detected by chemiluminescence using the ECL kit (GE Healthcare, Zurich, Switzerland). Bands of western blot were scanned and quantified by the Kodak 1D image analysis software.

### 2.9. Specificity and Sensitivity Calculations

Specificity and sensitivity were calculated by applying selected concentrations of tenascin-X to the serum from healthy patients (*n* = 7), patients with benign disease of the ovary (*n* = 8), and high-grade serous ovarian cancer patients (*n* = 13) and examining if they would be classified as positive or negative for ovarian cancer. The numbers of true positives (samples that tested positive and were cancerous (TP)), false negatives (sample that tested negative but were cancerous (FN)), false positives (samples that tested positive for ovarian cancer but were in fact benign or healthy tissue (FP)), and true negatives (samples that tested negative and were benign or healthy (TN)) were used to calculate the specificity and sensitivity as seen below:

(1) sensitivityTPTP+FN,specificity=TNFP+TN.

### 2.10. Statistical Analysis

Data were expressed as means ± SEM for *n* different samples. *P* values are calculated using Student's *t*-test and the *P* value < 0.05 was considered significant.

## 3. Results

### 3.1. Correlation between Tenascin-X and HSP10 with CA-125

Ascites from a single patient with FIGO stage IIIC serous adenocarcinoma were collected and purified from serum at four different points during treatment, denoted as A (obtained at the time of diagnosis), B (obtained after 3 cycles of paclitaxel/carboplatin and 1 cycle of gemcitabine), C (obtained at the end of the second cycle of gemcitabine), and D (obtained at the end of the third cycle of gemcitabine). We had previously determined a list of more than 60 proteins that showed differential expression in serous ovarian cancer cells compared to control and benign ovarian cells by proteomic analysis (submitted for publication). In this study, we examined the potential correlation between 32 of the most relevant of these proteins to the well-known and accepted ovarian cancer marker CA-125 by mass spectrometry. Two proteins, tenascin-X and HSP10, were found to have a strong positive correlation with CA-125 levels with Pearson's correlations of 0.999 and 0.990, respectively (Table 1). We have previously failed to confirm the potential of HSP10 to be a marker of ovarian cancer (submitted paper); thus, we only continued to examine the potential of tenascin-X as marker of ovarian cancer.

**Figure 1:**
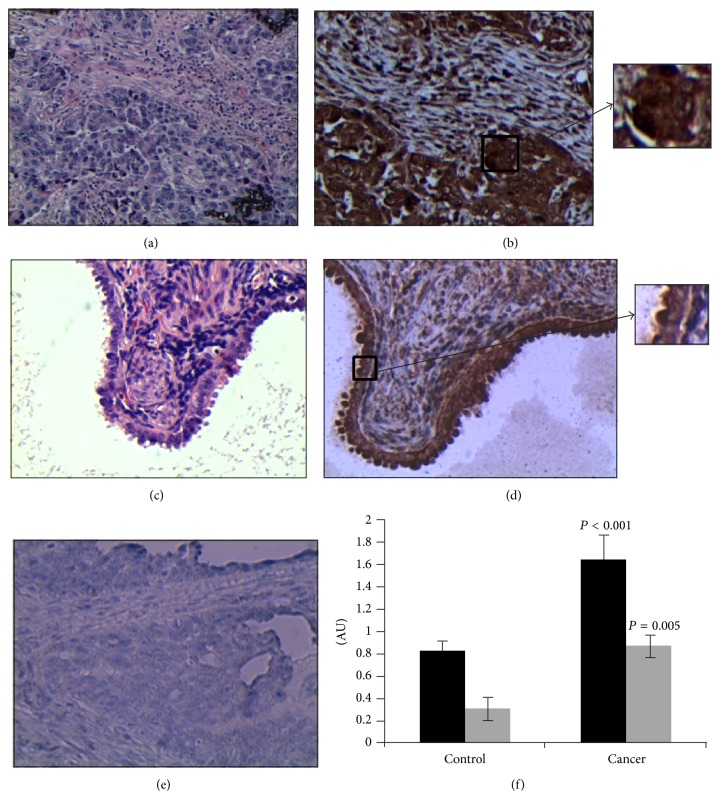
Expression of tenascin-X in ovarian healthy and cancerous tissue samples. (a) Hematoxylin eosin (HE) staining of ovarian cancer tissue. (b) Representative immunostaining of ovarian cancer tissue. (c) HE staining of ovarian tissue. (d) Representative immunostaining of healthy ovarian tissue. The magnification used is ×200. Square zones are enlarged 2-fold (resulting in magnification ×400). (e) Control of immunostaining. (f) The graph shows the score of staining intensity established by two experts with two different tenascin-X antibodies (in black: H-90, in grey: AF6999).  ^∗^ *P* value < 0.001; AU: arbitrary unit.

We next used immunohistochemistry to evaluate the presence of tenascin-X in both ovarian cancerous and control tissues. We found that tenascin-X is significantly more expressed in cancerous cells compared to ovarian healthy cells (Student's *t* value of 0.001, Figure 1).

### 3.2. Tenascin-X Level in Ovarian Cancer and Control Patients

**Figure 2:**
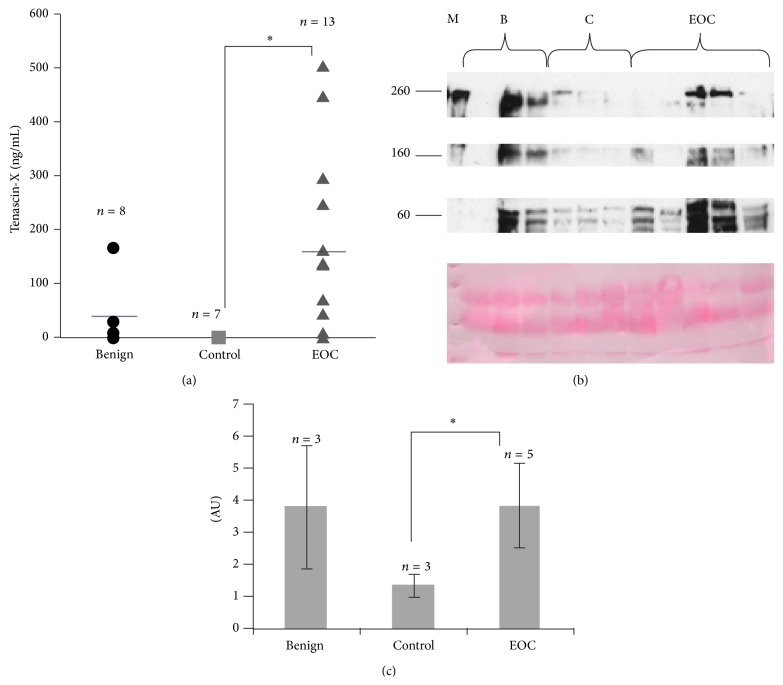
Tenascin-X serum levels in healthy, benign, and cancerous ovarian patients. (a) Tenascin-X levels were determined by ELISA assay following the manufacturer's instructions. ^∗^ *P* values < 0.05 were considered to be significant. (b) Representative western blot analysis of circulating tenascin-X and Ponceau S staining. M: molecular weight markers; B: benign; C: control; EOC: epithelial ovarian cancer. (c) Bands of western blot were scanned and quantified by the Kodak 1D image analysis software. The quantification was normalized to protein levels determined by Ponceau S staining. AU: arbitrary unit.

Due to the increased staining for tenascin-X in ovarian cancer tissue and the significant correlation between circulating CA-125 and tenascin-X secretion by ovarian cancer cells, we then evaluated tenascin-X level in serum of patients. Serum samples from high-grade serous ovarian cancer (*n* = 13), benign ovarian disease (*n* = 8), and healthy patients (*n* = 7) were collected and the levels of tenascin-X were determined by ELISA assay. We found that there was a significantly higher level of circulating tenascin-X in the serum of high-grade serous ovarian cancer compared to control patients group, which showed no expression of tenascin-X in any of the samples. Due to the low sample number and the wide variability in data in the benign group, the benign ovarian and control ovarian data were combined and compared to the high-grade cancer group. When the high-grade serous ovarian cancer group was compared to the combined control, there was a significantly larger amount of tenascin-X in serum of patients with high-grade serous ovarian cancer compared to healthy patients (Student's *t* value of 0.005, Figure 2).

Nevertheless, the level of tenascin-X in serum is lower than expected [12]. We thus decided to analyse tenascin-X in serum from 3 benign, 3 control, and 5 ovarian cancer serum samples by western blot analysis. Tenascin-X is a large protein of 450 kda, but different fragments of tenascin-X have already been identified in serum by western blot analysis. Depending on antibodies used, immunoreactive bands at around 250, 150, and 80 kDa have been already observed [13]. The 150 kDa tenascin-X species is a C-terminal fragment of full-length tenascin-X. At the opposite, the 250 kDa tenascin-X species is N-terminal fragment of tenascin-X.

Here, the western blot analysis of serum with sc-25717 antibodies gives the similar pattern of immunoreactive bands with FNIII27-28 antibodies [13]. Semiquantification of all immunoreactive bands (normalized to protein levels determined by Ponceau S staining) showed that tenascin-X is significantly more abundant in serum of ovarian cancer patients compared to controls (Figures 2(a) and 2(b)). In serum of benign patients, the level of tenascin-X is really different from one patient to the other one and globally it is not significantly different from cancer patients.

Due to the different forms of tenascin-X observed in serum of patients, it means that antibodies used for ELISA assay are particularly important. They may recognize all forms of tenascin-X or only tenascin-X species with C- or N-terminal part. This can explain, at least in part, the difference of tenascin-X level in control patients found in this report with other studies.

### 3.3. Tenascin-X and CA-125 in High-Grade Serous Ovarian Cancer Cells

**Figure 3:**
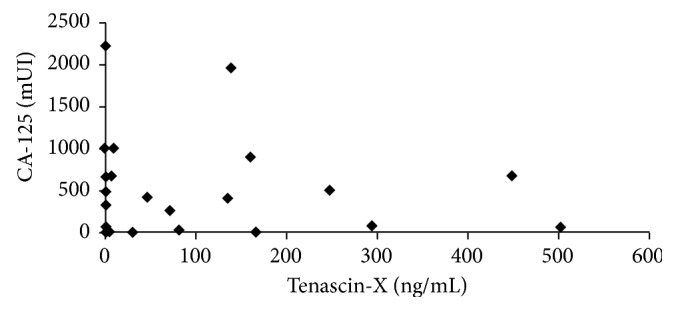
Correlation between tenascin-X and CA-125 levels in serum of benign and cancerous ovarian patients.

To test whether the positive correlation between CA-125 and tenascin-X in the initial patient study was true at the time of diagnosis in a larger population, we examined the level of CA-125 and tenascin-X in serum of healthy, benign ovarian cancer, and high-grade serous ovarian cancer patients by ELISA and correlated them (Figure 3). There was no correlation seen between the two proteins in serum.

### 3.4. Sensitivity and Specificity of Tenascin-X

**Figure 4:**
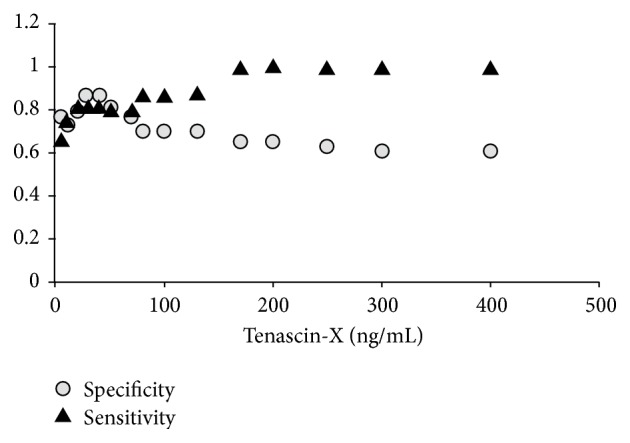
Specificity and sensitivity of tenascin-X in classifying ovarian cancer. Tenascin-X concentrations ranging from 0 to 400 ng/mL were applied to healthy, benign ovarian, and cancerous ovarian samples and classified as cancerous or healthy. Based on the number of true positives, true negatives, false positives, and false negatives, specificity and sensitivity were calculated as 0.87 and 0.82, respectively, with a cutoff value at 40 ng/mL.

For control, benign, and cancerous ovarian samples, concentrations of tenascin-X were chosen and applied to test the specificity and sensitivity of tenascin-X as a marker for ovarian cancer. The preliminary results based on our small collection of serum showed a specificity value of 0.87 and a sensitivity value of 0.82, with the highest values seen at a concentration of 40 ng/mL (Figure 4).

## 4. Discussion

The tenascin family is a highly conserved group of four large extracellular glycoproteins denoted as tenascin-C, -X, -R, and -W [14]. In most cells, the tenascin family interferes with the integrin-dependent spreading and affects cell motility and proliferation, often in contradicting ways. Indeed their role in cell proliferation seems to depend on the cell type. In some cell types they act as adhesive and promigratory, while in others they inhibit proliferation (for review see [15]). Tenascin-C is highly regulated in embryos as well as in adults, tenascin-R is expressed in the central nervous system where it is a major contributor to the brain ECM [16], and tenascin-W, which is the most recently described member, has been found in the bone [17]. Tenascins are potentially good diagnostic markers because they have limited distribution in healthy tissues [16]. However, only few studies reported the expression of tenascins in serous ovarian cancer. It was shown that expression of tenascin-C is increased in ovarian tumours compared with benign tumours and this may be associated with induction of specific isoforms [18]. It is predominantly secreted by fibroblasts and plays a role in adhesion and migration of ovarian cancer cells [19]. Based on these observations, Didem et al. recently investigated the clinical significance of the serum levels of tenascin-C in epithelial ovarian cancer patients [20]. Although serum level of tenascin-C is elevated in ovarian cancer patients, its predictive or prognostic role on survival in epithelial ovarian cancer patients seems to be not conclusive.

Tenascin-X is the largest, over 400 kDa, member and is widely expressed during development [21]. In adult tissue most of the expression of tenascin-X is seen in the connective tissue of the heart and skeletal muscle, as well as in the dermis. The tenascin-X gene is located in the major histocompatibility complex (MHC) in humans in a group of three other genes. This group of genes is collectively referred to as the RCCX and is repeated numerous times throughout the genome. Similar to the entire tenascin family, tenascin-X is composed of a cysteine-rich segment at the N-terminus, epidermal growth factor- (EGF-) like repeats, fibronectin III-like repeats, and a fibrinogen-like domain at the C-terminus [22].

The tenascin family has been seen to act differently depending on the microenvironment and the cell type examined [13]. In one study tenascin-X null mice proved that absence of tenascin-X enhances invasion and metastasis in melanoma cells while in another study it was shown that tenascin-X can bind to both isoforms of vascular epidermal growth factor- (VEGF-) B and enhance the ability of VEGF-B to stimulate endothelial proliferation [23, 24]. Its role in an organism is widely determined by the cell type that produces it and the microenvironment surrounding it.

Currently there are no reliable protein markers for the early diagnosis and the classification of ovarian cancer and due to the poor prognosis of late stage cancers there is an increasing need for markers to identify the cancer before metastasis. In a previous study comparing secretome of ovarian control and benign and cancer cells, we determined that tenascin-X is significantly differentially secreted by ovarian cancer cells. In this study we found that tenascin-X secretion by serous ovarian cancer cells purified from ascites taken during the follow-up of one patient had a strong positive correlation with circulating CA-125, suggesting that the secretion of CA-125 may correlate with the secretion of tenascin-X in the follow-up of patient. We then examined the expression of tenascin-X in both ovarian healthy and cancer tissues and saw a significant increased amount of tenascin-X protein in cancerous tissues compared to the control suggesting that it may be a useful marker of ovarian cancer. We next extended the study to compare tenascin-X levels in serum of healthy, benign disease, and ovarian cancer patients by ELISA assay. This revealed that serum from high-grade ovarian cancer patients had significantly more tenascin-X than control samples. mRNA level of tenascin-X has already been evaluated for its potential clinical value by analyzing the correlation between its expression and overall survival using publically available datasets (Supplementary Data, available online at http://dx.doi.org/10.1155/2015/208017). These datasets confirmed the potent interest in tenascin-X as marker of serous ovarian cancer.

Given the collection of evidence that tenascin-X may have conflicting roles in proliferation and metastasis depending on cell type and location and the high concentration of tenascin-X seen in high-grade ovarian cells, it is possible that high levels of tenascin-X may correlate with proliferation and metastasis of ovarian cancer.

We also saw that there was no correlation between CA-125 and tenascin-X levels in the different serums obtained at the time of diagnosis. This observation tends to suggest that CA-125 and tenascin-X may fluctuate in the same manner only during the follow-up of patients. We next examined the specificity and sensitivity of tenascin-X in correctly diagnosing ovarian cancer. We found that tenascin-X had a specificity of 0.87 and sensitivity of 0.82 with a cutoff value of 40 ng/mL. While these results are based on very preliminary results on a small sample number, the initial results are promising and hint that tenascin-X may be a useful marker for ovarian cancer.

Many efforts have been made to discover new biomarkers of ovarian cancer. However, all these new biomarkers do not perform better than CA-125 or other individual biomarkers, as observed for tenascin-X in this preliminary investigation. To improve predictive value of CA-125, it is combined with additional markers and defined as multiplexed biomarker approach. In this context, tenascin-C and tenascin-X could be integrated in the panel of biomarkers to be tested in multiplex biomarker panels [25].

## 5. Conclusion

Ovarian cancer is characterized by a poor prognosis due to a lack of accurate diagnostic tests. Here, we have shown that secretomic analysis is a suitable technique for the identification of protein biomarkers when combined with protein immunoassay and have identified tenascin-X as a potential marker for ovarian cancer. Based on preliminary results, tenascin-X may be a biomarker for ovarian cancer. It would be meaningful to extend this study to include a wider variety of samples at different stages to further substantiate tenascin-X as an ovarian cancer marker.

## Supplementary Material

Standard curve for increasing amounts of tenascin-X is shown (ELISA, Cusabio). We investigate the potential clinical value of tenascin-X using 3 different publically available datasets. PROGgene website was used to investigate the correlation of mRNA levels of tenascin-X survival and overall survival in ovarian cancer ()

## Figures and Tables

**Table 1:** Pearson's correlation between differentially secreted protein determined by LC-MS/MS (unique spectrum count) and circulating CA-125 in a follow-up patient study. Und = undetermined.

	A	B	C	D	Pearson's/CA-125
MMP-2	6	25	11	16	−0.616
Vimentin	10	13	17	12	−0.670
IGF binding	15	10	13	13	0.630
Gelsolin	3	5	5	2	−0.228
Thrombospondin-1	0	5	4	2	−0.744
Complement C3	134	72	79	104	0.802
Nucleobindin 1	9	8	6	6	0.853
DKK3	4	4	4	4	Und
IGF binding	14	11	8	18	0.117
CK8	27	13	25	16	0.613
Plasminogen activator inhibitor	35	19	33	29	0.463
MMP inhibitor 2	5	6	5	5	−0.205
Collagen alpha 2 (VI) chain	0	2	0	4	−0.559
CK7	26	4	7	15	0.812
Ezrin	20	7	13	15	0.679
Aldose reductase	16	7	13	7	0.755
Retinoic acid response	10	7	6	7	0.959
Complement factor B	41	26	33	46	0.212
Complement factor I	9	8	4	7	0.655
Sulfhydryl oxidase I	22	14	18	23	0.318
Fibrillin 1	6	0	2	3	0.788
Antileukoproteinase	9	2	19	17	−0.355
Endothelial protein C receptor	4	3	0	5	0.274
Beta-1,4-Galactosyltsferase 1	5	0	5	2	0.474
Calretinin	5	4	5	2	0.531
Carboxypeptidase A4	24	5	7	14	0.828
**Tenascin-X**	*12 *	*4 *	*3 *	*2 *	**0.999**
Agrin	22	14	15	2	0.768
Superoxide dismutase	4	3	7	2	0.003
Coiled-coil domain	6	9	6	5	−0.053
UTP-glc-1P uridylyl transferase	2	2	2	0	0.432
Laminin subunit	23	23	14	18	0.612
Matrix remodeling associated	6	10	10	3	−0.137
CK19	21	13	16	9	0.849
Alpha 2 macroglobulin	10	30	41	21	−0.751
**HSP10**	**2**	**0**	**0**	**0**	**0.990**
