# Supplementary material for: Secretome Identifies Tenascin-X as a Potent Marker of Ovarian Cancer
Source: Biomed Res Int. 2015 May 18;2015:208017. doi: 10.1155/2015/208017 (PMC4450242; doi:10.1155/2015/208017)

Supplementary data

Supplementary figure 1: *Standard curve for increasing amounts of tenascin X (ELISA, cusabio).*

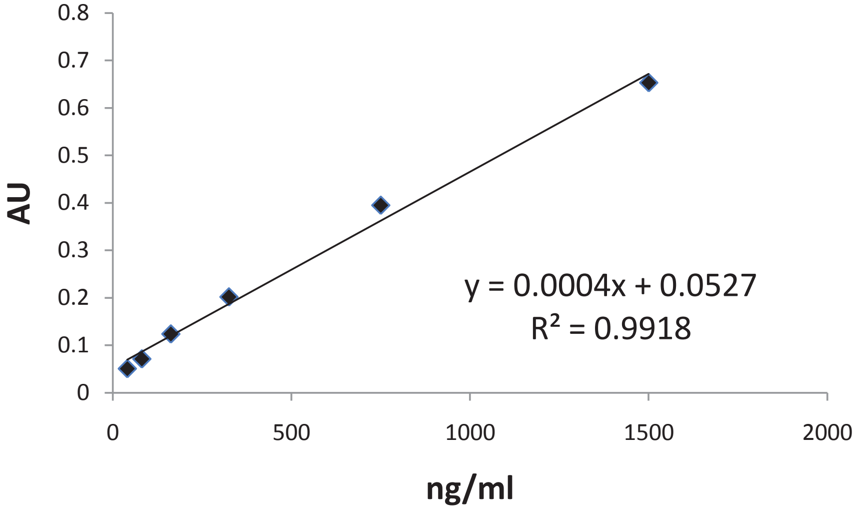

Supplementary figure 2: *Investigatation of the potential clinical value of tenascin X using 3 different publically available datasets.*

PROGgene website was used to investigate the correlation of mRNA levels of tenascin X survival and overall survival in ovarian cancer.

(http://watson.compbio.iupui.edu/chirayu/proggene/database/?url=proggene)

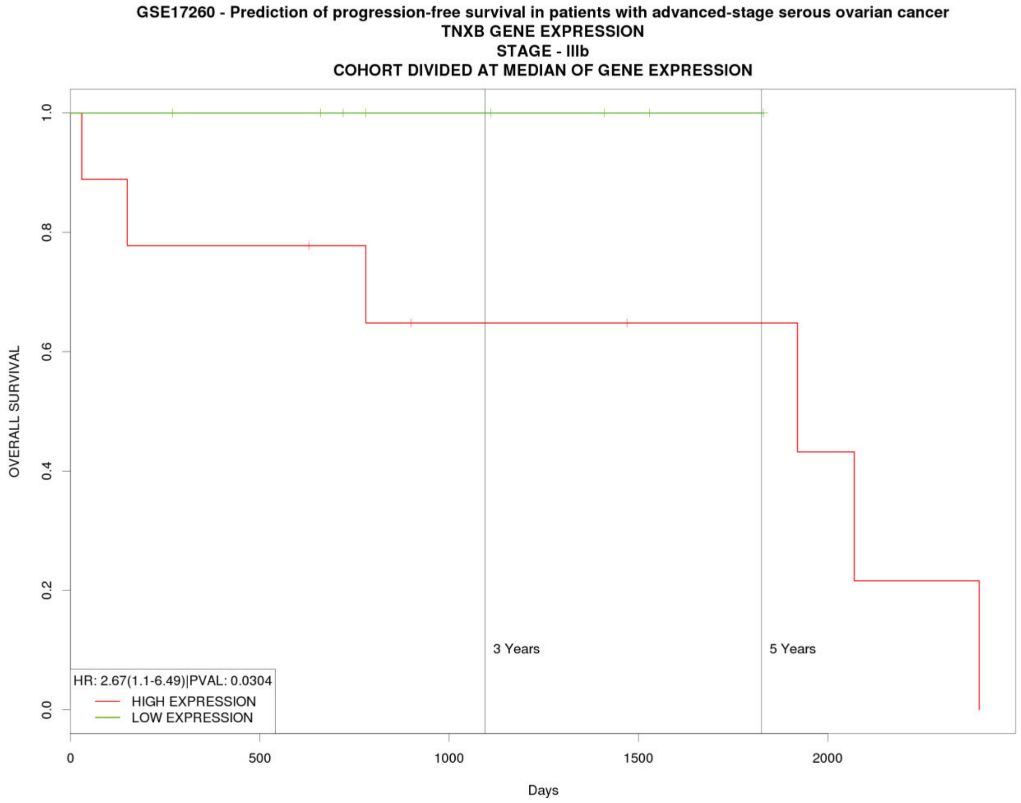

GSE26712 - A Gene Signature Predicting for Survival in Suboptimally Debulked Patients with Ovarian Cancer
TNXB GENE EXPRESSION
COHORT DIVIDED AT MEAN OF GENE EXPRESSION

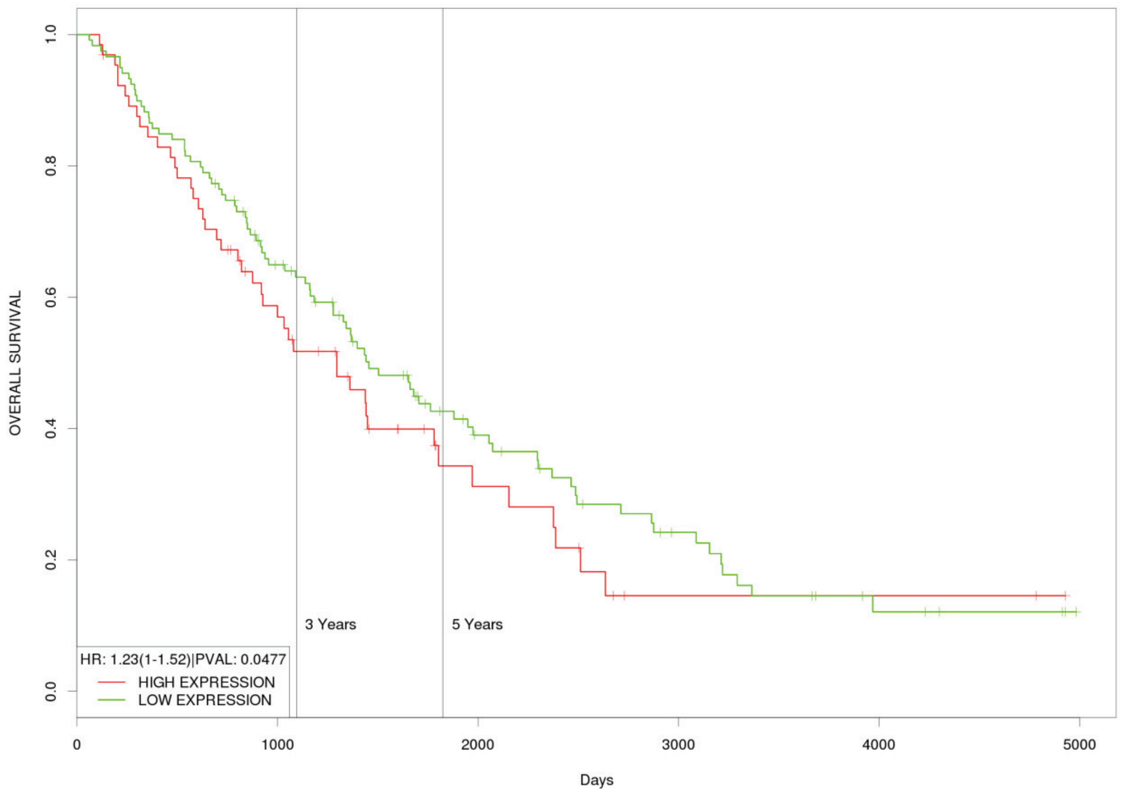

GSE17260 - Prediction of progression-free survival in patients with advanced-stage serous ovarian cancer
TNXB GENE EXPRESSION
STAGE - IIIb
COHORT DIVIDED AT MEAN OF GENE EXPRESSION

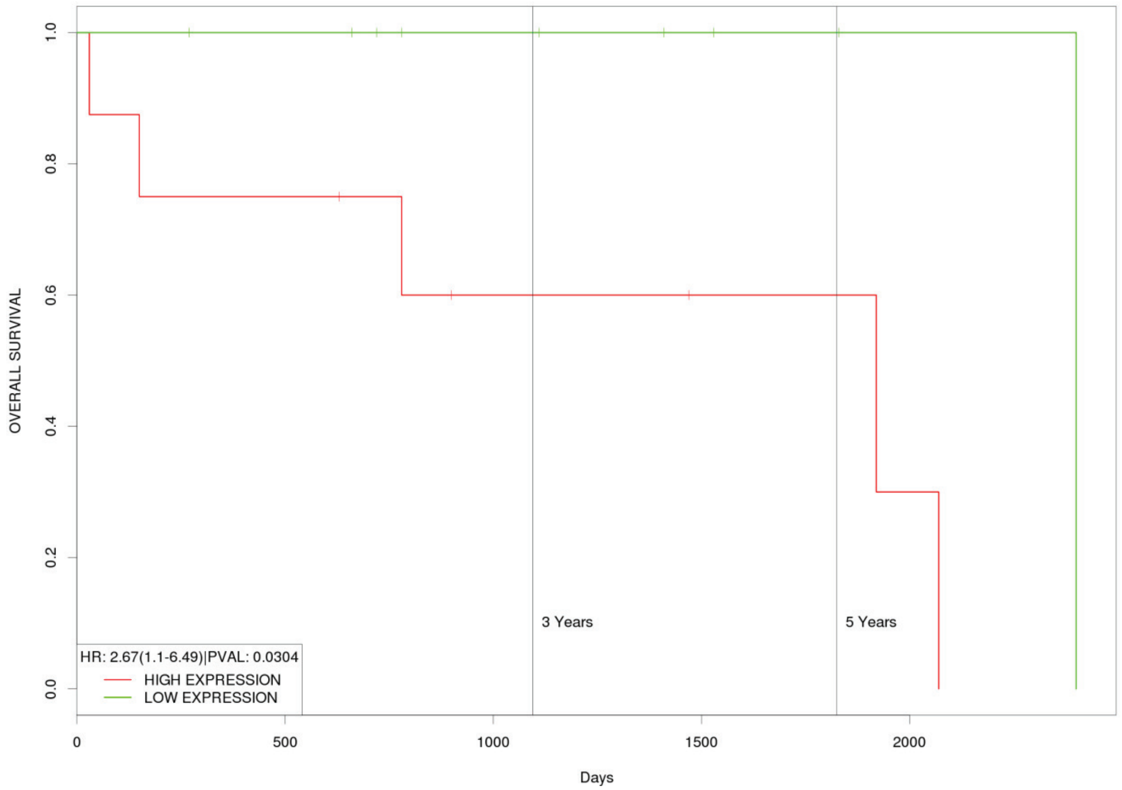

Supplement: Supplementary file 1 — Standard curve for increasing amounts of tenascin-X is shown (ELISA, Cusabio). We investigate the potential clinical value of tenascin-X using 3 different publically available datasets. PROGgene website was used to investigate the correlation of mRNA levels of tenascin-X survival and overall survival in ovarian cancer () [file 208017.f1.pdf]
